# Electrodeposition of Molybdenum Disulfide (MoS_2_) Nanoparticles on Monocrystalline Silicon

**DOI:** 10.3390/molecules27175416

**Published:** 2022-08-24

**Authors:** Martina Vizza, Walter Giurlani, Lorenzo Cerri, Nicola Calisi, Antonio Alessio Leonardi, Maria Josè Lo Faro, Alessia Irrera, Enrico Berretti, Juan Víctor Perales-Rondón, Alvaro Colina, Elena Bujedo Saiz, Massimo Innocenti

**Affiliations:** 1 Dipartimento di Chimica, Università degli Studi di Firenze, Via della Lastruccia 3, 50019 Sesto Fiorentino, Italy; 2 INSTM, Consorzio Interuniversitario Nazionale per la Scienza e Tecnologia dei Materiali, Via G. Giusti 9, 50121 Firenze, Italy; 3 Dipartimento di Ingegneria Industriale (DIEF), Università di Firenze, Via S. Marta 3, I-50139 Firenze, Italy; 4 Dipartimento di Fisica ed Astronomia, Università di Catania, Via Santa Sofia 64, 95123 Catania, Italy; 5 URT LAB SENS, Beyond Nano-CNR, c/o Department of Chemical, Biological, Pharmaceutical and Environmental Sciences, University of Messina, Viale Ferdinando Stagno d’Alcontres 5, 98166 Messina, Italy; 6 CNR-ICCOM, Istituto di Chimica dei Composti OrganoMetallici, Via Madonna del Piano 10, 50019 Sesto Fiorentino (FI), Italy; 7 Dipertimento di Chimica, Università di Burgos, Piazza Misael Bañuelos s/n, 09001 Burgos, Spain; 8 CSGI, Center for Colloid and Surface Science, Via della Lastruccia 3, 50019 Sesto Fiorentino, Italy

**Keywords:** MoS_2_, molybdenum disulfide, electrodeposition, monocrystalline silicon, nanoparticles, AFM, XPS, SEM, RBS

## Abstract

Molybdenum disulfide (MoS_2_) has attracted great attention for its unique chemical and physical properties. The applications of this transition metal dichalcogenide (TMDC) range from supercapacitors to dye-sensitized solar cells, Li-ion batteries and catalysis. This work opens new routes toward the use of electrodeposition as an easy, scalable and cost-effective technique to perform the coupling of Si with molybdenum disulfide. MoS_2_ deposits were obtained on *n*-Si (100) electrodes by electrochemical deposition protocols working at room temperature and pressure, as opposed to the traditional vacuum-based techniques. The samples were characterized by X-ray Photoelectron Spectroscopy (XPS), Scanning Electron Microscopy (SEM), Atomic Force Microscopy (AFM) and Rutherford Back Scattering (RBS).

## 1. Introduction

Molybdenum disulfide is a semiconductor that belongs to a family of layered transition-metal dichalcogenides (TMDCs). Recently, the applications of MoS_2_ have been widely investigated thanks to the superior physical and chemical properties of this material [1,2,3,4,5,6,7]. MoS_2_ is characterized by high light-absorbance capacity and abundance, low cost and a tunable band gap [6]. In fact, bulk MoS_2_ is an indirect semiconductor with a band gap of 1.2 eV [8], whereas its single-layered form shows a direct gap of 1.8 eV [6,9], enabling the production of switchable transistors [4,5] and photodetectors [4,10]. In particular, the 2D layered structure of MoS_2_ has interesting optical–electronic features that significantly differ from those in the bulk form [11,12]. Two-dimensional layered MoS_2_ has recently attracted great interest in the field of supercapacitors since it presents a sheet-like morphology and large in-plane conductivity. Therefore, this material is characterized by a high double-layer charge storage capacity and potential pseudocapacitive properties [5,13]. Additionally, the properties of MoS_2_ can be modified and adapted for a wide variety of different applications as a function of its morphology, particle dimensions and heterostructures [6]. In general, nanostructured MoS_2_ materials show great mechanical and opto-electronic properties, including excellent light-absorption and charge transfer features, as well as high wear resistance, toughness and mechanical friction. Recently, MoS_2_-based nanomaterials have attracted great interest in the field of dye-sensitized solar cells, Li-ion batteries and catalysis [6]. In particular, in 2005, Hinnemann et al. reported that MoS_2_ nanoparticles were active hydrogen evolution reaction (HER) catalysts [14,15]. The high activity, excellent stability and precious metal-free composition of MoS_2_ makes it suitable for performing sustainable and feasible HER electrocatalysis [16]. Different forms of active HER MoS_2_ electrocatalysts have been developed, including crystalline nanoparticles, amorphous films and molecular clusters [1,3,6,17,18,19,20,21,22,23,24,25,26,27]. MoS_2_ is also characterized by unique photocatalytic properties [3,6,28,29,30,31,32,33,34,35,36]. This is due to the fact that S-Mo-S coordination in the crystal lattice generates unsaturated Mo and S atoms at the edges, resulting in highly active sites to perform H_2_ production via water splitting [3,6,17,28,29,32,37]. In order to enhance the photocatalytic activity of MoS_2_ nanostructures, it is possible to couple them with semiconductors that enable rapid charge generation and reduce the combination rate of photoinduced electron-hole pairs [6]. Nowadays, silicon is one of the most important and widely used semiconductors, with various applications in electronics and photovoltaics and a great appeal for the realization of nanostructured materials [38,39,40,41,42,43]. Indeed, Si is an abundant material, presents strong visible light absorption and can be used in large-scale industrial production. Not only could Si be a promising candidate for water splitting applications [44,45,46], but its coupling with MoS_2_ could also be suitable for the future industrialized production of photo-electrodes [31,44,47,48]. MoS_2_ nanostructures can be prepared by means of top-down or bottom-up techniques. The first ones consist of etching crystal planes from a certain substrate, whereas in bottom-up approaches, the crystals are stacked over the substrate [1]. Examples of top-down techniques are micromechanical exfoliation [5,49], intercalation assisted exfoliation [42,50], solution exfoliation [51] and sputtering [52]. Bottom-up techniques include physical vapor deposition [53], hydrothermal synthesis [4], chemical vapour deposition [54] and atomic layer deposition [55]. However, all those techniques tend to be expensive and could not be easily extended to the industrial scale [4]. On the contrary, electrochemical deposition allows us to prepare nanoparticles and thin film materials at room temperature and pressure, still preserving a fine control on the morphology and thickness of the resulting material. In addition, electrodeposition could meet the demands for the feasible and industrial production of different materials [56], including molybdenum sulfides. Recently, the electrodeposition of MoS_2_ has been explored on a wide range of substrates [4,20,22,57,58,59]. As we previously pointed out, silicon could be an optimal substrate to expand the electrodeposition of MoS_2_ on a large scale. However, the electrodeposition of MoS_2_ on silicon has been little explored. This is mainly due to the semiconductor nature of Si, which is severely influenced by the lighting conditions and characterized by a limited exchange of electrons between the electrode and the solution. Therefore, if compared with metal working electrodes, finding the optimal conditions to perform electrodeposition on silicon electrodes could be very challenging [56]. For those reasons, in this article, we investigated the optimal conditions to obtain MoS_2_ on *n*-Si (100) using electrodeposition. We studied the electrochemical behavior of a tetrathiomolybdate solution as the precursor, and we prepared different samples by means of a potentiostatic and charge-controlled deposition technique. X-ray Photoelectron Spectroscopy (XPS), Scanning Electron Microscopy (SEM), Atomic Force Microscopy (AFM) and Rutherford Back Scattering (RBS) were used to characterize the obtained deposits. This work opens the route toward the use of electrodeposition as a cost-effective and scalable technique to couple MoS_2_ with silicon. Indeed, an electrochemical deposition technique able to work directly at room temperature and pressure was used, as opposed to traditional vacuum-based processes.

## 2. Results

**Figure 1:**
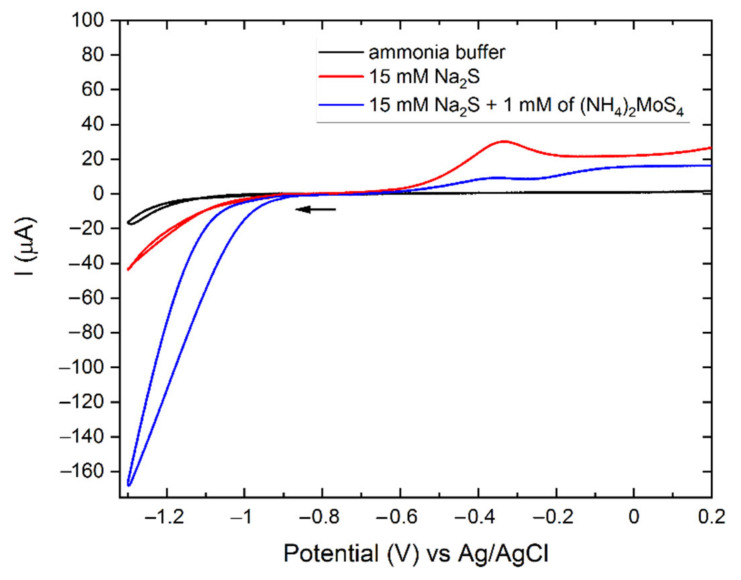
CVs of ammonia buffer (black), 15 mM Na_2_S in ammonia buffer (red), 1 mM (NH_4_)_2_MoS_4_ and 15 mM Na_2_S in ammonia buffer on *n*-Si (100) from –0.75 V to 0.2 V (blue), scan rate 10 mv/s.

The electrochemical behavior of the solutions is shown in Figure 1. The cyclic voltammetries (CVs) of the ammonia buffer and the 15 mM Na_2_S solution present low cathodic currents, which are compatible with the reduction of hydrogen. On the other hand, the CV scan of the deposition solution containing TTM shows a strong cathodic signal starting from −1.0 V and reaching –170 µA at –1.3 V. This cathodic current was attributed to the reduction of TTM to MoS_2_ (Equation (1)) [60].

(1) $${\mathrm{MoS}}_{4}^{2-}+2e^{-}+2H_{2}O\to {\mathrm{MoS}}_{2}+2{\mathrm{SH}}^{-}+2{\mathrm{OH}}^{-\;}.$$

No oxidative peaks were present during the anodic scan of the ammonia buffer solution. However, positive current values were registered from –0.75 V due to the gradual oxidation of the silicon electrode. The highest current value of 1.0 µA was recorded at the potential of +0.2 V (see Figure 1).

The anodic scan of the 15 mM Na_2_S solution shows higher positive currents starting from –0.6 V and the presence of a peak centered at –0.3 V, probably related to the oxidation of sulfide anions. Similar anodic behavior is also present in the CV of TTM. Therefore, we attributed this signal to the oxidation of S^2−^ instead of the oxidation of the MoS_2_ formed in the cathodic scan. An increase in the anodic current was present in the TTM solution at –0.1 V; this wide peak could be attributed to the partial oxidation of MoS_2_.

We prepared three different samples (A, B, C) using the deposition solution. We performed a potentiostatic charge-controlled deposition at three different potential values (–1.0 V; –1.1 V; –1.3 V). Independently from the applied potential, the total amount of deposited charge was 6 mC (Table 1). Every 200 µC, fresh solution was injected into the cell so that the concentration of the precursor on the surface of the electrode could remain constant. The procedure was repeated 30 times, performing 30 deposition cycles.

**Figure 2:**
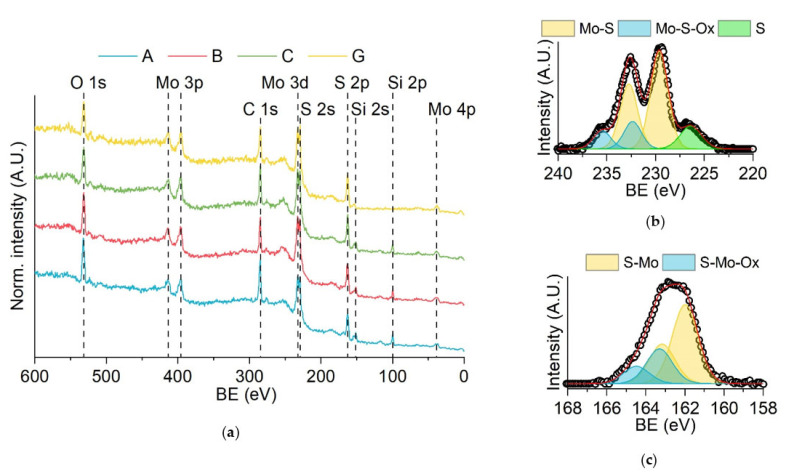
XPS spectra of the samples: (**a**) surveys of samples A, B, C and G; (**b**) high-resolution spectra of sample A in the Mo 3d region; (**c**) high-resolution spectra of sample A in the S 2p region.

The samples were characterized by XPS to evaluate the composition of the samples, determine the chemical state of each component and exclude the presence of contaminants. As expected, the survey spectra (Figure 2a) show the presence of sulfur and molybdenum, with additional peaks attributed to oxygen, carbon and silicon. The presence of oxygen in the samples can be related to oxidation phenomena, whereas the carbon signal is probably due to the adsorption of atmospheric carbon on the surface of the samples. Only the high-resolution spectra of sample A are shown in Figure 2b,c for explanatory purposes since they are very similar to the ones of samples B and C. Both the signals related to molybdenum and sulfur show two components. The principal one represents 70–80% of the total signal. The right ones (see the yellow doublets in Figure 2b,c) are located at energy values that are comparable with the ones previously reported for molybdenum disulfide (S 2p_3/2_ at 162.0 eV; Mo 3d_5/2_ at 229.6 eV) [61]. The second components on the left of the principal ones (the blue doublets in Figure 2b,c) can be attributed to local defects in the molybdenum disulfide lattice due to the presence of oxygen atoms. In the Mo 3d region, another peak at 226.4 eV is present, which was attributed to S 2s (green in Figure 2b). Taking everything into consideration, we can say that the change in the deposition potential in the range between –1.0 V and –1.3 V did not affect the final composition of the deposit. 

We prepared four new samples (D, E, F, G) using a deposition potential of –1.1 V and varying amounts of deposited charge (2 mC, 6 mC, 18 mC and 36 mC). Every 200 µC, fresh solution was injected into the cell to keep the concentration of the precursor on the surface of the electrode constant. This procedure was repeated a certain number of times so that different deposition cycles were performed: 10, 30, 90 and 180 (Table 2).

XPS analysis was also performed on sample G (see Figure 2) to evaluate any change in the molybdenum sulfide deposit compared with samples A–C. Molybdenum disulfide was also detected in sample G, with local defects in the lattice due to the presence of oxygen atoms. No significant differences could be noticed between samples A, B, C and G. Therefore, the increase in the number of deposition cycles did not affect the final composition of the deposit.

**Figure 3:**
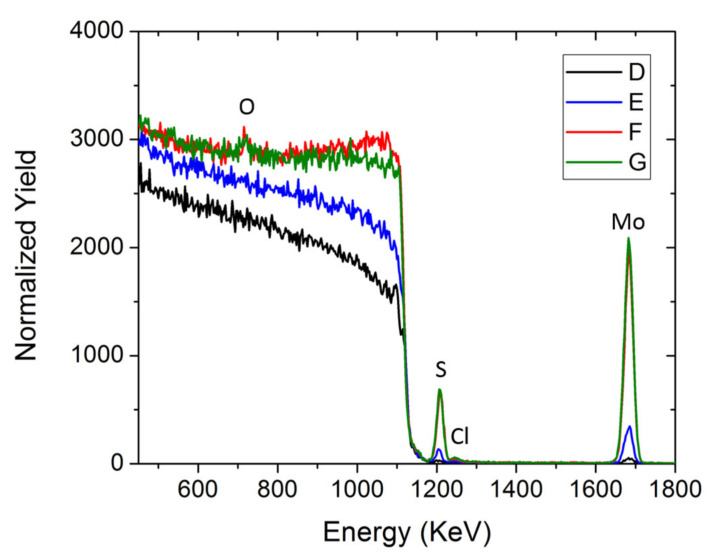
RBS spectra of the D, E, F and G samples.

The sample compositions were obtained from the simulation fit of their RBS spectra, as reported in Figure 3. Measuring the integrated area, it is possible to estimate the elemental atomic surface density per element (at/cm^2^), hence the MoS_x_ stoichiometry reported in Table 3 for each sample. The RBS spectra also report the presence of O, probably due to the partial oxidation of the sample that was previously confirmed by XPS analysis. Moreover, Figure 3 show minor chloride contamination, probably due to the leakage of the reference electrode. The increment in the elemental surface density increases linearly for the first cycles, whereas at higher cycling, it tends to saturate all the analyzed elements. Consequently, the stoichiometry slightly differs from sample to sample. In particular, the S/Mo ratio varies from 2.52 ± 0.15 in sample D to 1.89 ± 0.11 in sample G.

**Figure 4:**
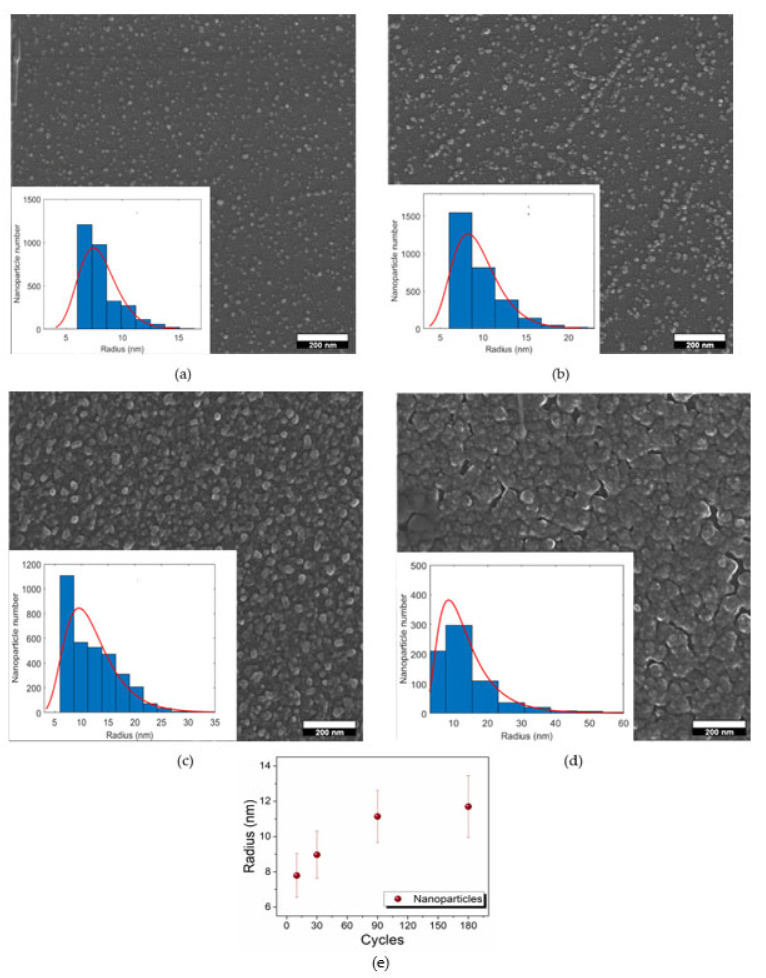
SEM images of: (**a**) sample D, 10 cycles; (**b**) sample E, 30 cycles; (**c**) sample F, 90 cycles; (**d**) sample G, 180 cycles; (**e**) correlation between the dimensions of the nanoparticles and the number of the deposition cycles. The insets of figures a-d show the statistical analysis of the dimension of the nanoparticles performed on the SEM images.

SEM characterization was performed on the D–G samples to obtain information about the morphology of the deposits and the electrode coverage. Figure 4 show the presence of nanoparticles in all the samples, with different distributions on the surface of *n*-Si (100). Both the dimensions and the coverage of the electrode surface change from one sample to the other. For a better understanding of the morphology of the deposit, statistical analysis was performed on the SEM images of the D–G samples (insets in Figure 4). ImageJ software version 1.x (National Institutes of Health, MD, USA) was used, and thousands of nanoparticles were analyzed. Since the SEM images were acquired after the metallization of the samples, only the nanoparticles with a radius higher than 5 nm were considered. Histograms were fitted using a lognormal curve due to the asymmetrical distribution of the nanoparticles. The median value of the radius of the nanoparticles was about 8 ± 1 nm and 9 ± 1 nm in samples D and E, respectively. The average radius increased up to the value of about 11 ± 1.5 nm in sample F, and 12 ± 2 nm in sample G. Figure 4e show the correlation between the dimensions of the nanoparticles and the number of deposition cycles. The average radius of the nanoparticles almost linearly increased with the number of cycles in samples D–F, whereas the dimensions of the nanoparticles in samples F and G were very similar. However, it must be noted that only circularly shaped nanoparticles were considered to perform statistical analysis on sample G, consequently discarding bigger aggregates on the surface of the electrode. The SEM images show that the coverage of the electrode surface gradually increased from sample D to sample G. Indeed, samples D and E were characterized by the presence of nanoparticles spotted on the surface of the substrate. The number of nanoparticles on the electrode was higher in sample F, whereas in sample G, the coalescence between nanoparticles was predominant, and a continuous film was almost produced. Therefore, the coverage of the electrode surface was minimum inthe case of the samples obtained with a lowest number of cycles (10, 30), increased moving to the 90-cycle sample (F) and was maximum in the case of the 180-cycle sample (G).

**Figure 5:**
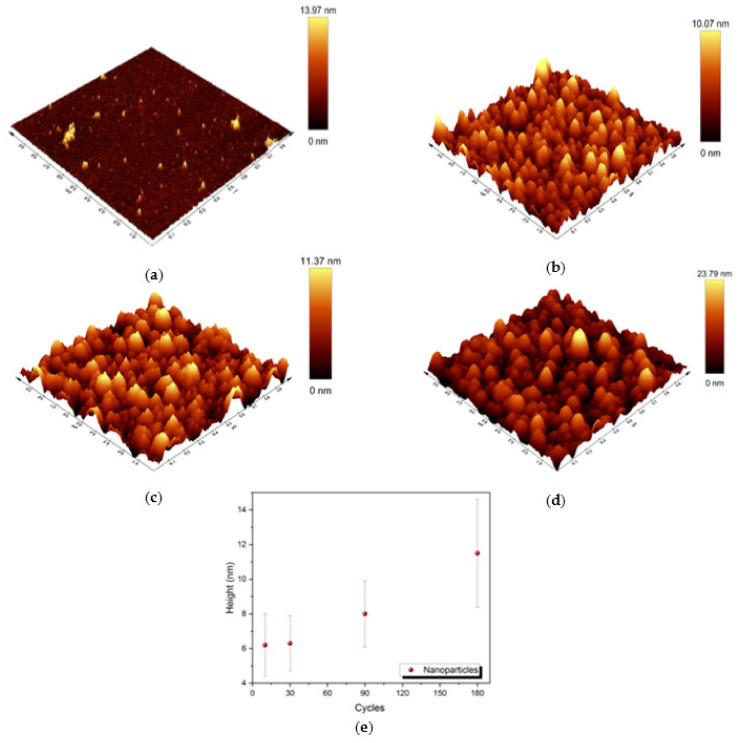
Three-dimensional view of 1 µm × 1 µm AFM images of samples: (**a**) D; (**b**) E; (**c**) F; (**d**) G; (**e**) correlation between the medium height of the nanoparticles and the number of deposition cycles in samples D–G.

For a better understanding of the morphology of the samples, AFM characterization was performed (see Figure 5). The medium height of the nanoparticles was about 6.2 ± 1.8 nm, 6.3 ± 1.6 nm, 8.0 ± 1.9 nm and 11.5 ± 3.1 nm in samples D, E, F and G, respectively. The correlation between the height of the nanoparticles and the number of deposition cycles is shown in Figure 5e. Considering the standard deviation, the average height obtained with AFM is in line with the average radius obtained by SEM, confirming the presence of almost spherical-shaped nanoparticles on top of the Si substrate.

## 3. Materials and Methods

### 3.1. Electrochemical Measurements

Sigma Aldrich ammonium tetrathiomolybdate (TTM) (NH_4_)_2_MoS_4_ and sodium sulfide Na_2_S were used without further purification. 0.1 M ammonia buffer (pH = 9.2) was the basis of all the solutions and was prepared by mixing Merck analytical reagent grade NH_4_OH 28%, HClO_4_ 65% and MilliQ water (18 MΩ, Merk Millipore, Burlington, MA, USA). We prepared a solution containing 15 mM Na_2_S and the deposition solution by mixing 1 mM (NH_4_)_2_MoS_4_ and 15 mM Na_2_S in the ammonia buffer. Na_2_S excess in the deposition solution served both as an S precursor and to prevent the precipitation of insoluble MoO_2_ [20,60]. The solutions were deaerated with nitrogen and stored under a nitrogen atmosphere in sealed Pyrex jars. A PC-controlled automated system (University of Florence, Florence, Italy) was used to perform electrodeposition [62]. The capacity of the cell was 1.88 mL. The working electrode was developed from *n*-Si 100 (*p*-doped with a resistivity of 1–5 Ω∙cm), exposing a circular area with a diameter of 1 cm. The electrode was cleaned following the RCA procedure prior to each measurement [56]. The electrochemical depositions were carried out at room temperature in the dark to exclude the influence of light that could potentially lead to the photoexcitation of silicon. All the potential values refer to the Ag/AgCl sat. KCl reference electrode.

### 3.2. Microscopic and Spectroscopic Characterization

Scanning electron microscopy (SEM) images were acquired using a Gaia 3 manufactured by Tescan (Brno, Czech Republic) with a detector for secondary electrons. The energy was equal to 20 kV for image acquisition. The composition of the MoS_2_ deposit was evaluated by X-ray photoelectron spectroscopy (XPS). The samples were fixed to the sample holder using double-side graphitic tape and then transferred into the XPS ultra-high vacuum (UHV) chamber. The XPS instrumentation makes use of a non-monochromatic X-ray source (VSW Scientific Instrument Limited model TA10, Manchester, UK), Mg Kα radiation, 1253.6 eV), operating in this case at 144 W (12 kV and 12 mA), and of a hemispherical analyzer (VSW Scientific Instrument Limited model HA100, Manchester, UK). The analyzer was equipped with a 16-channel detector and dedicated differential pumping system that permitted it to work during the acquisition with pressure in the main chamber up to the 10^−8^ mbar range. The pass energy was set to 44 eV. The obtained spectra were analyzed using CasaXPS dedicated software (version 2.3.19, Casa Software Ltd., Teignmouth, UK). The inelastic background was subtracted by means of Shirley’s method [63], whereas mixed Gaussian and Lorentzian contributions were used for each component. The lowest component relative to the 1s transition of carbon for adventitious carbon was shifted to 284.8 eV in order to calibrate the spectra [64]. Rutherford Backscattering Spectrometry (RBS) was carried out using a He^+^ beam at an energy of 2 MeV. The backscattered He^+^ ions were collected at the detection angle of 165° with respect to the beam direction, and their energy loss was determined by a multichannel analyzer. The RBS spectra were fitted using SIMNRA 7.03 simulation software (Max-Planck-Institut für Plasmaphysik, Garching, Germany). AFM measurements were performed with tapping-mode on an atomic force microscope model Alpha300A AFM (Witec, Ulm, Germany) under ambient conditions. All images were obtained using the commercial cantilever type Arrow^TM^ FMR forced modulation type (AC) with reflex coating (reflex aluminum), premounted on a magnetic ring.

## 4. Conclusions

In this article, we investigated the possibility to electrodeposit molybdenum disulfide on *n*-Si (100). Initially, we studied the electrochemical behavior of 1 mM (NH_4_)_2_MoS_4_ and 15 mM Na_2_S in ammonia buffer (pH = 9.2) as the deposition solution. Then, we prepared different deposits by means of potentiostatic charge-controlled electrodeposition and varying the applied potential. XPS analysis confirmed the presence of molybdenum disulfide in all the investigated samples, with local defects related to the presence of oxygen atoms. The change in the deposition potential values (between −1.0 V and −1.3 V) did not affect the final composition of the deposits. New samples were prepared at −1.1 V with a different number of deposition cycles (from 10 to 180). With RBS measurement, we evaluated the stoichiometric ratio between Mo and S, confirming the XPS results. SEM and AFM characterizations showed the presence of nanoparticles in all samples. The average height and radius values of the molybdenum disulfide nanoparticles increased with the number of deposition cycles, reaching the highest values of 11.5 ± 3.1 nm and 12 ± 2 nm, respectively, in the 180-cycle sample (G). Additionally, the electrode coverage increased with the number of the deposition cycles, with coalesced nanoparticles forming an almost continuous film in sample G. Taking everything into consideration, this paper opens the route towards the production of MoS_2_ nanoparticles on monocrystalline silicon using electrodeposition. This easy, low-cost and scalable technique working directly at room temperature and pressure, opens the route towards the integration of MoS_2_ production in large-scale silicon-manufacturing processes.

## Figures and Tables

**Table 1:** Deposition parameters of samples A, B and C.

Sample	Deposition Potential	Total Deposited Charge	Number of Cycles	Deposited Charge/Cycle
A	–1.0 V	6 mC	30	200 µC
B	–1.1 V	6 mC	30	200 µC
C	–1.3 V	6 mC	30	200 µC

**Table 2:** Deposition parameters of samples D, E, F and G.

Sample	Deposition Potential	Total Deposited Charge	Number of Cycles	Deposited Charge/Cycle
D	–1.1 V	2 mC	10	200 µC
E	–1.1 V	6 mC	30	200 µC
F	–1.1 V	18 mC	90	200 µC
G	–1.1 V	36 mC	180	200 µC

**Table 3:** Surface density per element (at/cm^2^) and MoS_x_ stoichiometry calculated from the RBS measurements.

Sample	S 10 ^15^ at/cm^2^	Mo 10 ^15^ at/cm^2^	O 10 ^15^ at/cm^2^	S/Mo
D	2.14 ± 0.06	0.85 ± 0.03	--	2.52 ± 0.15
E	8.80 ± 0.26	4.48 ± 0.13	--	1.96 ± 0.12
F	39.90 ± 1.20	23.1 ± 0.69	32.55 ± 0.98	1.73 ± 0.10
G	47.62 ± 1.43	25.17 ± 0.75	40.04 ± 1.20	1.89 ± 0.11

## Sample Availability

Samples of the compounds are available from the authors.
